# Peripheral dose from megavoltage cone‐beam CT imaging for nasopharyngeal carcinoma image‐guided radiation therapy

**DOI:** 10.1120/jacmp.v13i5.3869

**Published:** 2012-09-06

**Authors:** Ming X. Jia, Xu Zhang, Na Li, En Y. Wang, Da W. Liu, Wei S. Cai

**Affiliations:** ^1^ Department of Radiation Oncology Shengjing Hospital of China Medical University, Shenyang Shenyang China; ^2^ The Fourth Hospital of China Medical University Shenyang China

**Keywords:** megavoltage bone‐beam CT, nasopharyngeal carcinoma IGRT, peripheral dose

## Abstract

The growing use of cone‐beam computed tomography (CBCT) for IGRT has increased concerns over the additional radiation dose to patients. The in‐field dose of IGRT and the peripheral dose (PD) from kilovoltage CBCT (KV‐CBCT) imaging have been well quantified. The purpose of this work is to evaluate the peripheral dose from megavoltage CBCT (MV‐CBCT) imaging for nasopharyngeal carcinoma IGRT, to determine the correlation of peripheral dose with MU protocol and imaging field size, and to estimate out‐of‐field organ‐at‐risk (OAR) dose delivered to patients. Measurements of peripheral MV‐CBCT doses were made with a 0.65 cm^3^ ionization chamber placed inside in a specially designed phantom at various depths and distances from the imaging field edges. The peripheral dose at reference point inside the phantom was measured with the same ionization chamber to investigate the linearity between MUs used for MV‐CBCT imaging and the PD. The peripheral surface doses at the anterior, lateral, and posterior of the phantom at various distances from the imaging field edge were also measured with thermoluminescent dosimeters (TLDs). Seven nasopharyngeal carcinoma patients were selected and scanned before treatment with head–neck protocol, and the peripheral surface doses were measured with TLDs placed on the anterior, lateral, and posterior surfaces at the axial plane of 15 cm distance from the field edge. The measured peripheral doses data in the phantom were utilized to estimate the peripheral OAR dose. Peripheral dose from MV‐CBCT imaging increased with increasing number of MUs used for imaging protocol and with increasing the imaging field size. The measured peripheral doses in the phantom decreased as distance from the imaging field edges increased. PD also decreased as the depth from the phantom surface increased. For the patient PD measurements, the anterior, lateral, and posterior surface doses of 15 cm distance from the field edge were $2.84\times 10^{-2}$, $1.01\times 10^{-2}$, and $0.78\times 10^{-2}\;\text{cGy/MU}$, respectively. The lens, thyroid, breast, and ovary and testicle, which are outside the treatment and imaging fields, were estimated to receive peripheral OAR doses from MV‐CBCT imaging of $42.4\times 10^{-2}$, $11.9\times 10^{-2}$, $1.4\times 10^{-2}$, $1.0\times 10^{-2}$, and $0.5\times 10^{-2}\;\text{cGy/MU}$, respectively. In conclusion, MV‐CBCT generates a peripheral dose beyond the edge of the MV‐CBCT scanning field that is of a similar order of magnitude to the peripheral dose from kV‐CBCT imaging. In clinic, using the smallest number of MUs allowable and reducing MV‐CBCT scanning field size without compromising acquired image quality is an effective method of reducing the peripheral OAR dose received by patients.

PACS number: 89

## I. INTRODUCTION

The technique of intensity modulated radiation therapy (IMRT) has been widely used in the clinical environment to treat nasopharyngeal carcinoma, providing a steep dose gradient between tumor and surrounding critical organs, and leading to the potential for higher dose to be delivered to the target while sparing normal tissues.^1^, ^3^ However, the clinical application of IMRT requires increasing accuracy in patient setup and treatment delivery to ensure that the steep gradient dose distribution is maintained. This is where image‐guided radiation therapy (IGRT) plays a key role.^4^, ^9^

To date, two CBCT techniques for IGRT, kilovoltage CBCT (kV‐CBCT) and megavoltage CBCT (MV‐CBCT), have been developed to verify a patient's setup accuracy. Both are able to generate a three‐dimensional anatomical dataset for a patient in treatment position. The kV‐CBCT system consists of a kV X‐ray tube and a radiographic detector mounted on the gantry of a linear accelerator. MV‐CBCT uses the existing parts on the linear accelerator — the treatment beam and the electronic portal imaging device — to create the three‐dimensional image, thus reducing cost, simplifying QA, and allowing for a full integration into the treatment workflow.^10^, ^12^

The in‐field and out‐of‐field doses delivered by the kV‐CBCT imaging system have been recently reported in the literature^13^, ^16^ while, to date, only in‐field dose has been discussed for the MV‐CBCT system.^17^, ^25^ Morin et al.^(17)^ reported the image acquisition dose delivered to patients from MV‐CBCT imaging for 5 and 9 MU protocols on pelvis and head‐and‐neck patients. They also evaluated the physical performance and image optimization of MV‐CBCT.^(20)^ Gayou et al.^(21)^ reported the patient dose and image quality from MV‐CBCT imaging for seven different MU protocols, (3, 5, 8, 10, 12, 15, and 60 MU), as well as the commissioning and clinical implementation of the MVision system.^(22)^ Peng et al.^(24)^ calculated the patient dose resulting from the MV‐CBCT and orthogonal pair techniques for six treatment sites. Pouliot et al.^(25)^ evaluated the image quality of MV‐CBCT using low‐dose, and further demonstrated how the MV‐CBCT system can be applied for patient alignment. For the kV‐CBCT system, Islam et al.^(13)^ reported point doses at various depths in a cylindrical water phantom. Ding and Coffey^(14)^ calculated the dose to organs from a kV‐CBCT imaging guidance procedure using the VMCBC algorithm. Kan et al.^(15)^ performed dosimetric measurements using a female anthropomorphic phantom with thermoluminescent dosimeters for the OBI system, and reported the effective doses to the body and the absorbed doses to 26 organs using the standard mode and the low‐dose mode.

Peripheral dose (PD) is of clinical interest in estimating detriment to organs sensitive to relatively low doses of radiation. Such detriments include the risk of cataract formation to the lens of the eye, as well as the risk of carcinogenesis particularly to the thyroid and breast.^26^, ^28^ The concept of PD is normally associated with IMRT treatment, due to the increasing number of monitor units used. IGRT using CBCT imaging techniques result in an even greater additional dose being delivered to the patient, and there is a larger volume of normal tissue that is irradiated by the low radiation dose. The CBCT imaging peripheral dose is usually defined as dose anywhere outside the imaged area. Perks et al.^(16)^ reported that peripheral dose from kV‐CBCT needs to be taken into account when considering long‐term care of radiation oncology patients because peripheral dose contributed by CBCT can be on the same order of magnitude as the IMRT peripheral dose. However, no data have been published to date specifically for the MV‐CBCT peripheral dose.

The purpose of this study was to evaluate the peripheral dose from MV‐CBCT imaging in head‐and‐neck IGRT, to investigate the impact of different MU protocols and imaging field sizes on the peripheral dose, and to estimate the out‐of‐field organs at risk (OAR) dose from MV‐CBCT imaging for nasopharyngeal carcinoma IGRT treatment.

## II. MATERIALS AND METHODS

### A. MV‐CBCT imaging system

Measurements of the MV‐CBCT peripheral dose were carried out on a linear accelerator (ONCOR, Siemens, Germany) equipped with an amorphous silicon flat panel (AG9‐ES, PerkinElmer, Optoelectronics). The MV‐CBCT acquisition is similar to an arc treatment. The linear accelerator gantry rotates in a continuous 200° arc (270° to 110°, clockwise) acquiring one portal image per degree to reconstruct a three‐dimensional image. The maximum field size is $27.4\times 27.4\;{\text{cm}}^{2}$, with the field width fixed at 27.4 cm, while the field length is adjustable to a maximum value of 27.4 cm. The number of monitor units (MU) used for MV‐CBCT imaging is specified by the operator and ranges from 2 to 60 MU. Usually, 5–15 MU protocols are used in the clinical setting, and the 60 MU protocol is reserved for calibration purposes.

### B. Water phantom measurements

**Figure 1:**
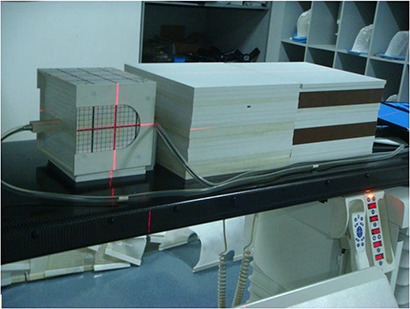
Solid water slab phantom used to measure peripheral doses.

A test phantom was created from three sets of solid water slabs (Fig. 1) to simulate head and neck cases. The head of the phantom consisted of $20\times 20{\;\text{cm}}^{2}$ rectangular solid water stacked to an anterior–posterior depth of 20 cm. The trunk of the phantom was two sets of $30\times 30{\;\text{cm}}^{2}$ solid water slabs stacked to a depth of 20 cm, lined up longitudinally to a length of 60 cm. Two slabs of $12\times 10{\;\text{cm}}^{2}$ solid water were inserted between head and trunk to represent the neck, 4 cm in length.

The out‐of‐field dose in the phantom from MV‐CBCT imaging with various MU protocols was measured using a $0.65\;{\text{cm}}^{3}$ cylindrical ionization chamber (IBA, Inc, Germany) in order to verify linear relationship between the peripheral dose and MUs used for MV‐CBCT imaging. The phantom was positioned on the treatment couch so that the center of its head coincided with the machine isocenter, and so that the longitudinal axis of the phantom was perpendicular to the plane of gantry rotation. The ionization chamber was placed 10 cm below the phantom's surface at the median plane (isocenter vertical plane), at a distance of 15 cm from the position of the geometrical field edge in the plane of measurement. The phantom was then scanned by MV‐CBCT system with 5, 8, 15 and 30 MU protocols and a $27.4\times 27.4\;{\text{cm}}^{2}$ field size. Three measurements were taken for each protocol.

The MV‐CBCT peripheral dose at different locations for different field sizes was measured with the same ionization chamber in the same phantom. Three measurements were taken for each measurement location. First, measurements were carried out at three different depths of 1, 5, and 10 cm from the phantom's surface at the median plane, with an 8 MU protocol for $27.4\times 27.4\;{\text{cm}}^{2}$ field size. For each depth, 10 measurement locations were established at distances of 1, 3, 5, 10, 15, 20, 25, 30, 35, and 40 cm from the geometric field edge. Secondly, the same measurements were made for a field size of $27.4\times 27.4\;{\text{cm}}^{2}$ at depths of 1 and 10 cm. Next, the doses at two pair of points (9 cm from median line, at anterior–posterior and left–right directions) were measured in the axial plane 5 cm from the field edge to investigate the homogeneity of peripheral dose distribution. Finally, thermoluminescent dosimeters (TLDs) were used to measure peripheral dose profiles away from the field edge at the anterior, lateral, and posterior surfaces of the phantom for the 8 MU protocol and $27.4\times 27.4\;{\text{cm}}^{2}$ field size.

The ionization chamber used in this study was calibrated by the dosimetry calibration laboratory of the National Institute of Standards using a standard ${}^{60}\text{Co}$ beam energy. The inherent uncertainty was ±0.27%, the reproducibility was ±0.13%. The total uncertainty associated in our dosimeter was estimated to be within ±2.0% in this study. The TLDs used in this study were selected from the same batch and calibrated in the low dose range (0–0.2 Gy) with reproducibility within ±3%, and dose detection ranging from $10^{-7}\;\text{Gy}$ up to 12 Gy. The TLDs were read using the FJ‐427A1 reader (BNI, Inc, China). The total uncertainty associated in our TLDs system was estimated to be within ±8.9% (κ=2) for the low‐dose ranges.

### C. Patient measurements

In addition to the phantom measurements, MV‐CBCT peripheral dose measurements in nasopharyngeal carcinoma radiotherapy patients were performed. A total of seven nasopharyngeal cancer patients with mean age 45 years (range 19–65) were treated using a seven‐field step‐and‐shoot IMRT technique with prescribed dose of 70 Gy in 35 fractions on a Siemens ONCOR linear accelerator. During a treatment period of one week, MV‐CBCT imaging was performed twice for each patient, leading to a total of 14 MV‐CBCT scans (5 MU for 11 scans and 8 MU for the remainder). For each MV‐CBCT scan, the Y jaws of the linac were moved as close as possible in the superior–inferior (SI) direction to establish an imaging field that was approximately 4 cm larger (2 cm each superiorly and inferiorly) than the SI extension of PTV, resulting in a reduction of peripheral dose to OARs.

The MV‐CBCT PD was measured in patients with TLDs. The TLDs were placed at the anterior, lateral, and posterior surface positions of the patients at the axial plane 15 cm away from the field edge. The protocol and field size settings for the MV‐CBCT imaging were recorded for every patient, and the distances from the field edge and depth of the OAR (lens, thyroid, breast, ovary, and testes) were measured and estimated. The OAR dose to patients was estimated by measuring the depth and distance from the field edge of the OAR in the body and performing calculations based on the measurement data in the phantom.

### D. Statistical analysis

The Pearson product‐moment correlation coefficient was used to measure the correlation between MUs from MV‐CBCT imaging and peripheral doses. The Wilcoxon‐Mann‐Whitney test was used to compare the PD difference for different depths and field sizes. All tests were performed using SPSS, version 13.0 (SPSS Inc., Chicago, IL)

## III. RESULTS

### A. Linearity between peripheral dose and MUs

**Figure 2:**
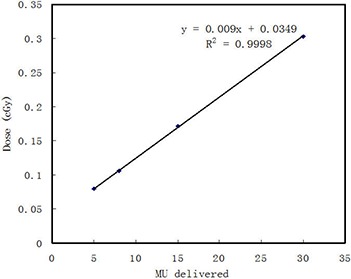
Dose delivered at 15 cm out‐of‐field distance at 10 cm depth by the 5, 8, 15, and 30 MU protocols in the phantom. Data were fitted with a linear function.

Figure 2 shows measured peripheral doses for $27.4\times 27.4\;{\text{cm}}^{2}$ field size and 5, 8, 15, and 30 MU protocols. The data demonstrate a strong linear relationship between peripheral dose

and MUs used for MV‐CBCT imaging $\left(R^{2}=0.9998\right)$. Increasing the MUs will result in a higher peripheral dose.

### B. Slab phantom peripheral dose

**Figure 3:**
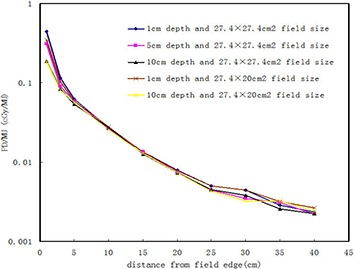
Out‐of‐field PD/MU in the phantom at depths of 1, 5, and 10 cm for the $27.4\times 27.4\;{\text{cm}}^{2}$ field size, and at depths of 1 and 10 cm for the $27.4\times 27.4\;{\text{cm}}^{2}$ field size.

Figure 3 shows the measured peripheral dose (defined as PD/MU) from MV‐CBCT imaging in head‐and‐neck region as a function of the distance from the field edge at depths of 1, 5, and 10 cm for the $27.4\times 27.4\;{\text{cm}}^{2}$ field size, and at depths of 1 and 10 cm for the $27.4\times 27.4\;{\text{cm}}^{2}$ field size. Each point on the curve was a mean value of three measurements, with a standard deviation of less than 0.01 cGy/MU. As demonstrated in the figure, the PD/MU decreased almost exponentially with increasing distance of measurement. The measured PD/MU decreased with increasing depth of measurement, but the decrease varied little with increasing distance from the field edge. For example, at a distance of 1 cm, the measured PD/MU at depths of 1, 5, and 10 cm were 0.45, 0.31 and 0.19 cGy/MU, respectively. At a distance of 5 cm, they were 0.063, 0.059, and 0.054 cGy/MU, respectively. The test results indicated no significant differences in the PD/MU among the depths of 1, 5, and 10 cm (p>0.05). The measured PD/MU increased with increasing MV‐CBCT scan field size, and the increase varied with depth. When the distance from field edge was 1 cm at the depth of 1 cm, an increase in field size of 37% resulted in a PD/MU increase of 28%. However, at same distance of 1 cm and at the depth of 10 cm, the PD/MU increased only 1.8%. The test results indicated no significant differences in the PD/MU between the $27.4\times 27.4\;{\text{cm}}^{2}$ and $27.4\times 27.4\;{\text{cm}}^{2}$ field size (p>0.05).

**Figure 4:**
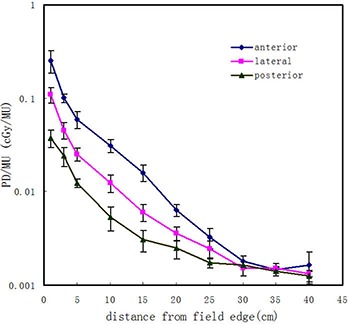
Out‐of‐field PD/MU at anterior, lateral, and posterior surfaces of the phantom for the $27.4\times 27.4\;{\text{cm}}^{2}$ field size.

Figure 4 shows the PD/MU profiles away from the field edge at the anterior, lateral, and posterior surfaces of the phantom for the $27.4\times 27.4\;{\text{cm}}^{2}$ field size. The results indicated that the peripheral dose at the anterior surface of the phantom was always higher as compared to the corresponding peripheral dose at the lateral and posterior surface positions.

The peripheral doses of the four direction locations (anterior, posterior, left, and right) in the phantom axial plane 5 cm from the field edge were $6.31\times 10^{-2}$, $4.38\times 10^{-2}$, $4.97\times 10^{-2}$, and $5.01\times 10^{-2}\;\text{cGy/MU}$, respectively, with a standard deviation of less than $0.02\times 10^{-2}\;\text{cGy/MU}$. These measurements confirm that peripheral dose anteriorly is greater than the dose at the other directions, reflecting the heterogeneity of peripheral dose distribution from the MV‐CBCT imaging.

### C. Patient peripheral dose

For patient peripheral dose measurements, the measured peripheral surface dose at anterior, lateral, and posterior surfaces of 15 cm axial plane from the field edge were $2.84\times 10^{-2}$, $1.01\times 10^{-2}$, and $0.78\times 10^{-2}\;\text{cGy/MU}$, respectively, with standard deviations of $0.32\times 10^{-2}$, $0.27\times 10^{-2}$, and $0.11\times 10^{-2}\;\text{cGy/MU}$.

The MV‐CBCT scan parameters and measurement parameters, such as field size and OAR depth and distance from the field edge, are summarized in Table 1. These data were utilized to estimate the OAR peripheral dose in nasopharyngeal carcinoma IGRT. The average doses to lens, thyroid, breast, ovary, and testes were estimated at $42.4\times 10^{-2}$, $11.9\times 10^{-2}$, $1.4\times 10^{-2}$, $1.0\times 10^{-2}$, and $0.5\times 10^{-2}\;\text{cGy/MU}$, respectively.

**Table 1:** MV‐CBCT scan field length (Y jaws in SI direction), depth, and distance from the field edge for the OAR in each patient.

				*Depth/Distance of OAR (cm)*			
*Patient*	*Age/Sex*	*Field Length (cm)*	*Lens*	*Thyroid*	*Breast*	*Ovary*	*Testes*
P1	46/M	19.2	0.5/1	2.0/2	0.5/15		3/62
P2	65/M	17.6	0.5/2	2.0/4	0.5/16		3/60
P3	28/F	16.0	0.5/1	2.0/3	3.0/15	7/34	
P4	19/M	20.6	0.5/1	2.5/5	0.5/16		3/65
P5	52/M	18.0	0.5/2	2.0/4	0.5/15		3/61
P6	61/F	16.5	0.5/1	2.0/3	2.0/18	9/31	
P7	42/M	22.4	0.5/1	2.5/5	0.5/16		3/63

## IV. DISCUSSION

The growing use of MV‐CBCT for improving the accuracy of patient setup and tumor localization in IGRT has increased concerns over the associated additional in‐field and peripheral radiation dose associated with the procedure. A number of data about in‐field dose from MV‐CBCT imaging have been published,^10^, ^17^, ^18^, ^20^, ^24^ but no data about peripheral dose from MV‐CBCT imaging have been reported. Therefore, it is useful to determine the peripheral dose from MV‐CBCT imaging.

Peripheral dose is usually expressed as a percentage of maximum dose at the central axis in the field, or it is correlated to the prescription dose. For MV‐CBCT imaging, peripheral dose depends strongly on the MU protocol used for imaging. Therefore, it is more convenient to describe peripheral dose from MV‐CBCT as PD/MU.

The results of the present study have shown that the MV‐CBCT peripheral dose decreases almost exponentially with increasing distance from the field edge. Moreover, PD increases with increasing CBCT scan field size, and decreases with increasing measurement depth. Though test results show no significance for different depths and field sizes, our measuring results show the PD varied obviously with depth, and field size at 1 cm depth within 5 cm distance from the field edge. Some studies have been reported the PD from the MV treatment field.^29^, ^31^ These studies have looked at this phantom and found very little depth dependence for the measured PD — a result that is different from ours, possibly it is because there are only 200° of arc delivery from 270° to 110° for the MV‐CBCT imaging process. Another result of the measurement was that the PD was heterogeneous, with higher dose at the anterior than the posterior. This indicates that the PD from the MV‐CBCT may have a particular depth‐dependence.

Perks et al.^(16)^ reported a measured PD of 0.2 cGy at 25 cm from the central axis of an anthropomorphic phantom in the kV‐CBCT scan at 120 kV and $26\times 26\;{\text{cm}}^{2}\;\text{cm}$ field size. In this study we obtained a comparable peripheral dose of 0.18–0.20 cGy at 11.3 cm away from field edge (equivalent to 25 cm from the central axis of phantom) for MV‐CBCT imaging with an 8 MU protocol and $27.4\times 27.4\;{\text{cm}}^{2}$ field size. MUs as low as 5 may be used for MV‐CBCT imaging, to result in a reduction of peripheral dose.

Uncertainties in measurements of the PD should be ±9% or less, based on repeated measurements. The contribution to the overall uncertainty due to possible errors in the measurement location is not included. Uncertainties in estimation of the OAR doses are likely to be much greater because of the difficulty in measuring accurately OAR location in the body and estimating accurately OAR dose using a same phantom measurement data for the bodies of different individual patients. Uncertainties in the OAR doses were conservatively estimated to be ±50% or less.

This study has also shown that in nasopharyngeal carcinoma IGRT, lens and thyroid will receive relatively high peripheral doses because they are located at or near the patient surface and close to the edges of the scanning field. Effective methods for decreasing peripheral dose in these organs may include using as small a scan field size as possible without sacrificing image quality, moving Y jaws close to each other to shield these organs, and performing prone MV‐CBCT scans and treatments. This study should be useful for the clinical application of MV‐CBCT by providing MV‐CBCT peripheral dose estimations and effective methods for peripheral dose reduction in MV‐CBCT scans. In clinical settings where a high MU protocol is required to obtain sufficient soft‐tissue contrast, the imaging field size should be decreased accordingly to avoid high peripheral dose.

## V. CONCLUSIONS

Knowledge of peripheral dose from MV‐CBCT imaging is necessary for radiation oncologists to determine MV‐CBCT scan frequency and scan parameters. In this study, MV‐CBCT imaging resulted in peripheral doses comparable to kV‐CBCT imaging. The peripheral dose due to MV‐CBCT imaging, although small compared to the dose from the MV treatment beam, may be significant if the MV‐CBCT procedure is applied daily and, therefore, should be taken into account when planning IGRT using MV‐CBCT technology.
